# Generation of Concentration Gradients by a Outer-Circumference-Driven On-Chip Mixer

**DOI:** 10.3390/mi13010068

**Published:** 2021-12-31

**Authors:** Fumiya Koike, Toshio Takayama

**Affiliations:** Department of Mechanical Engineering, Tokyo Institute of Technology, 2-12-1, Ookayama, Tokyo 152-8552, Japan; takayama.t.aa@m.titech.ac.jp

**Keywords:** micromixer, microfluidics, density control, lab on a chip, pneumatically driven

## Abstract

The concentration control of reagents is an important factor in microfluidic devices for cell cultivation and chemical mixing, but it is difficult to realize owing to the characteristics of microfluidic devices. We developed a microfluidic device that can generate concentration gradients among multiple main chambers. Multiple main chambers are connected in parallel to the body channel via the neck channel. The main chamber is subjected to a volume change through a driving chamber that surrounds the main chamber, and agitation is performed on the basis of the inequality of flow caused by expansion or contraction. The neck channel is connected tangentially to the main chamber. When the main chamber expands or contracts, the flow in the main chamber is unequal, and a net vortex is generated. The liquid moving back and forth in the neck channel gradually absorbs the liquid in the body channel into the main chamber. As the concentration in the main chamber changes depending on the pressure applied to the driving chamber, we generated a concentration gradient by arranging chambers along the pressure gradient. This allowed for us to create an environment with different concentrations on a single microchip, which is expected to improve observation efficiency and save space.

## 1. Introduction

Traditionally, cell-culture and chemical-reaction experiments are conducted in containers whose size is similar to that of a Petri dish. However, a Petri dish is used for a very small observation target, and for the aforementioned experiments, a large space is required, as much waste fluid is generated. To solve these problems, research on microfluidic devices and microreactors was conducted to realize a cell culture and chemical mixing on microchips by scaling down experiments that were conducted at a laboratory scale [1,2,3,4,5,6,7].

As most microfluidic devices process a low Reynolds number, liquids are mixed using molecular diffusion, which is very time-consuming. To solve this problem, various methods have been proposed, and they can be classified into passive and active mixers. Passive mixers are designed to increase the contact area of two liquids by applying ingenuity to the shape of the flow path, thereby promoting diffusion [8,9,10,11]. For instance, Chien-Chong Hong et al. studied mixing using a Tesla valve [12], and Sung-Jin Park studied mixing using a three-dimensional flow channel [13]. Active mixers use electrophoresis, ultrasonic or other waves to forcibly generate a vortex for mixing [11,14,15,16]. Ahmed et al. proposed a mixing process based on the ultrasonic vibration of microbubbles [17]. Glasgow et al. proposed a mixing method based on pulsed flow [18].

To observe the behavior of cells in various environments on a microfluidic device, it is necessary to realize various concentrations on a microchip. There are two ways to achieve this: one is to use diffusion to generate a concentration gradient and keep supplying it [19,20,21,22], and the other is to prepare multiple chambers with different concentrations [23,24,25,26,27,28]. With regard to the former method, Fukuda et al. succeeded in supplying a stable concentration gradient by using a branching channel and a meandering channel [29]. For the latter, Hung et al. succeeded in creating multiple chambers with different concentrations on a microchip by orthogonally flowing the concentration gradient-generated liquid and perfusion liquid on the microarray [30]. Bo Dai et al. realized a continuously varying concentration gradient in the side chamber of the body channel just through the flow of solution [31].

**Figure 1:**
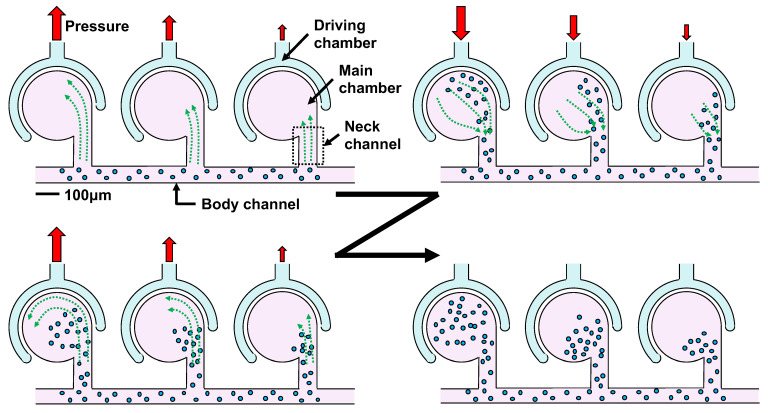
Driving principle of outer-circumference-driven mixer. The liquid in the main channel is gradually siphoned into the main chamber. Mixing speed can be changed by driving pressure.

The authors developed an active mixer (outer-circumference-driven mixer) that uses the inequality of flow during the expansion or contraction of chambers [32,33,34,35,36]. The driving principle of the outer-circumference-driven mixer is shown in Figure 1. The outer-circumference-driven mixer comprises a main chamber that mixes the two liquids, a driving chamber that surrounds the main chamber, a body channel through which the liquid flows, and a neck channel that connects the main chamber to the body channel. When pressure is applied to the driving chamber, the main chamber expands and contracts through the elastic wall. When this process is repeated, the liquid in the body channel is gradually absorbed into the main chamber with a slight back and forth movement through the neck channel. This repeated expansion or contraction mixes with the liquid in the main chamber and reaches the concentration in the body channel. When the main chamber reaches the desired concentration, air can be injected into the body channel to maintain the concentration in the main chamber.

The mixing speed of this mixer is proportional to the amplitude of the liquid moving back and forth in the neck channel, and the amplitude of the neck channel is proportional to the expansion or contraction of the main chamber. In this study, we generated a concentration gradient by applying a pressure gradient to each driving chamber to differentiate the volume of the main chamber. If we can create chambers with different concentrations on a single microchip, we can save space, reduce the amount of waste fluid, and improve the observation efficiency.

## 2. Materials and Methods

### 2.1. Experimental Method and Channel Design

In a previous study [32,33,34,35,36], a piezoelectric actuator was used to drive the driving chamber through water; however, in this study, air pressure was used. The transmission channel to the driving chamber was filled with water, and the water was vibrated using a piezoelectric actuator. The piezoelectric actuator can powerfully drive the driving chamber because it can force the volume change; however, the volume change is limited. Therefore, as the number of main chambers increases, the volume change supplied to the driving chamber decreases, and there is a concern that the same results cannot be obtained when the number of chambers is small. Since a drive source such as a piezoelectric actuator is required to vibrate water at high speed, it is difficult to achieve a large scale with water in the transmission channel. Therefore, we decided to use air pressure, because it is possible to increase the pressure even when there is a large number of main chambers.

**Figure 2:**
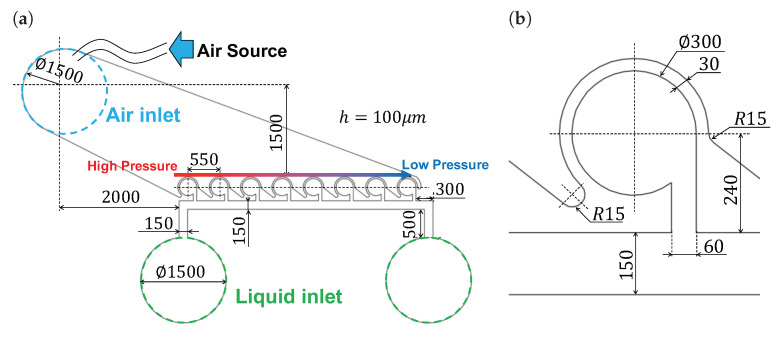
Design of the microchip. (**a**) Overall dimensions of flow channel. Air pressure is supplied from the air source. Arrow above the driving chamber indicates the expected pressure supplied to the driving chamber. (**b**) Detailed dimensions of the main chamber, which were determined on the basis of previous studies [33].

As the absorption rate of the main chamber can be adjusted by the pressure applied to the driving chamber, the concentration gradient can be generated by arranging chambers along the pressure gradient. The designed channel is shown in Figure 2. The process of why this channel design was chosen is described in Appendix A. A tube was connected to the air inlet, as shown in Figure 2a, a solenoid valve was connected to a tube, and a compressor was connected to a solenoid valve. Moreover, the syringe was connected to the liquid inlet to supply the liquid to body channel. The farther the distance from the air pressure source is, the narrower the flow path at the top of the driving chamber becomes. Therefore, the narrower the channel is, the more difficult it is to transmit pressure, and concentration gradients can thus be generated.

In Figure 2b, the main chamber dimensions were determined on the basis of previous studies [33]. In this study, we used the same dimensions, but there is room for debate as to whether the dimensions are suitable for using air pressure. Determining the optimal dimensions of the main chamber when air pressure is used is a future task.

To confirm the principle of this mixer, that is, whether flows are different between expansion and contraction, we conducted simulations using Autodesk CFD2020. Since this mixer is driven by the wall deformation at high frequency, it is difficult to simulate the flow caused by it. Therefore, we assumed that pressure is applied to the wall of the main chamber and verified whether the streamlines are different during expansion and contraction.

**Figure 3:**
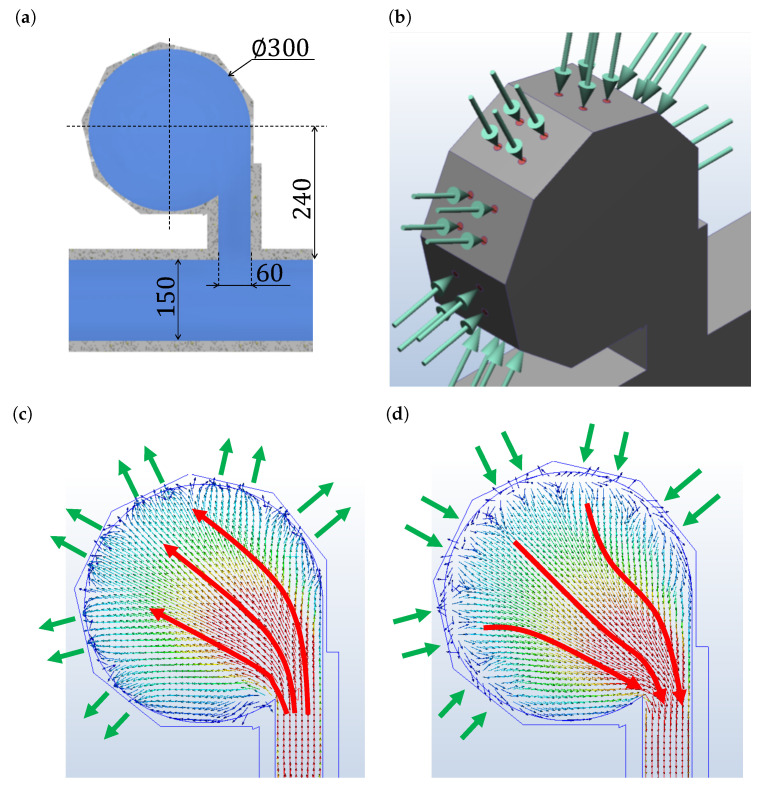
Simulation results. (**a**) Main chamber model. Because the surface to which pressure is applied must be flat, the outer shape was polygonal. (**b**) How to apply pressure to the main chamber. Since it was not possible to simulate the change in flow owing to the deformation of the wall, it was assumed that pressure is applied. (**c**,**d**) Streamlines during expansion and contraction. Streamlines were different between expansion and contraction; therefore, the net vortex was created by repeating this process.

The result of simulation is shown in Figure 3. Figure 3a denotes the model, Figure 3b denotes how to apply the pressure, and Figure 3c,d denote the results that are the streamlines of expansion and contraction. Since the surface to which pressure is applied must be flat, the flow channel outline was polygonal. The streamlines were different between expansion and contraction, confirming the inequality of flows, which is the principle of this mixer.

### 2.2. Microchip Fabrication

A silicon wafer was spin-coated with SU8-3050 (MicroChem Inc., Japan), and prebaked (95 °C, 45 min). A mask with a channel pattern was placed on SU8 and irradiated with UV light (exposure energy, 250 mJ/cm^2^, 10 s). After that, a mold with the channel pattern was developed with a thinner. Polydimethylsiloxane (PDMS, SILPOT184, Dow Inc., Japan, base:curing agent = 9:1 (mass ratio)) was poured into the mold. After deaeration and curing, PDMS that was transferred into the channel pattern was bonded to the slide glass using plasma treatment to create a flow channel.

### 2.3. Experimental Setup

**Figure 4:**
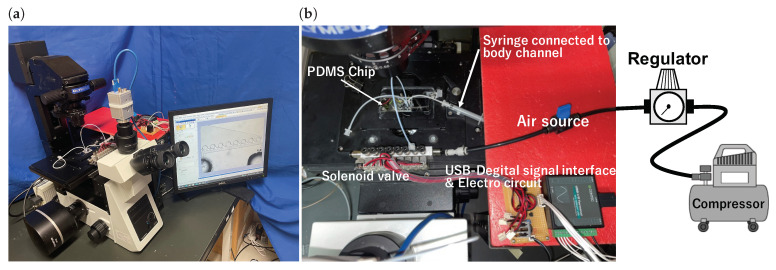
(**a**) Experimental setup. (**b**) Experimental apparatus.

Figure 4 shows the experimental apparatus and microchip drive unit. The air was supplied from the compressor (AK-T20R, Max Co., Ltd., Japan.) to the solenoid valve, which was controlled by the microcomputer (AIO-160802AY-USB, CONTEC Co., Ltd., Japan) through the regulator. This air was supplied to the driving chamber, and the air pressure causes a volume change in the driving chamber. The main chamber was filled with pure water, and a mixture of 3 μm microbeads (Polybead Polystyrene 3.0 Microspheres, Polysciences Inc., Philadelphia, PA, USA) and pure water flowed in the body channel. We used 3 μm microbeads to improve the streamlines more easily to see and increase the contrast for easier analysis.

In this study, air pressure was 0.30 MPa, the solenoid valve operated at 50 Hz, and duty ratio was 50%. This condition was experimentally determined to maximize absorption speed. Although there may be more optimal conditions by adjusting frequency, pulse width, etc., we used this condition in the experiment. The exploration of the ideal parameters is a future task.

### 2.4. Evaluation

**Figure 5:**
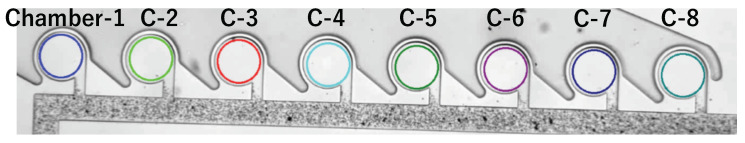
Measured concentration. Numbering from left to right is 1, 2, …, 8. Calculations were performed using Python and OpenCV.

The concentration was quantitatively evaluated using the luminance value. We recorded the experiment with a microscope (OLYMPUS, IX73P1F, Japan), measured the change in luminance value of each main chamber on the basis of the program, and evaluated the concentration using the following formula:

(1) $$L_{n}\left(t\right)=\sum^{A_{n}}\frac{Pxl(t,x,y)}{S_{n}}$$

(2) $$MI=\frac{L_{n}\left(t\right)}{L_{n}\left(0\right)}$$

where MI denotes the mixing index, *n* denotes the number of main chambers, $A_{n}$ denotes the area of the *n*-th chamber, $S_{n}$ denotes the number of pixels in $A_{n}$, and Pxl(t,x,y) denotes the pixel value. The concentration of each main chamber was measured in the area shown in Figure 5. Moreover, Python and OpenCV were used to calculate the luminance values and measure the concentrations at each frame.

We produced three chips and performed three experiments using each chip. We evaluated whether the concentration gradient was generated on the basis of MI in the nine experiments. The chamber that was supplied with high pressure absorbed and agitated the liquid. The more the chamber absorbed the liquid, the lower the luminance value and consequently the lower the MI were. Therefore, the lower the MI was, the more absorption and agitation occurred in the chamber.

## 3. Result

**Figure 6:**
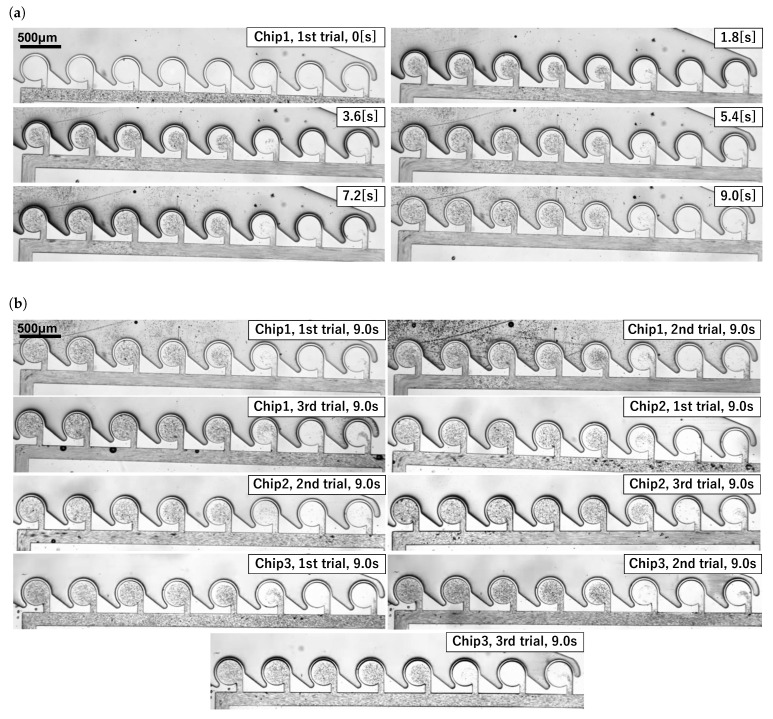
Generation of concentration gradient using an outer-circumference-driven mixer. (**a**) Time variation of an experiment (Chip 1, first trial). (**b**) Experimental results. Similar results were obtained from nine experiments.

**Figure 7:**
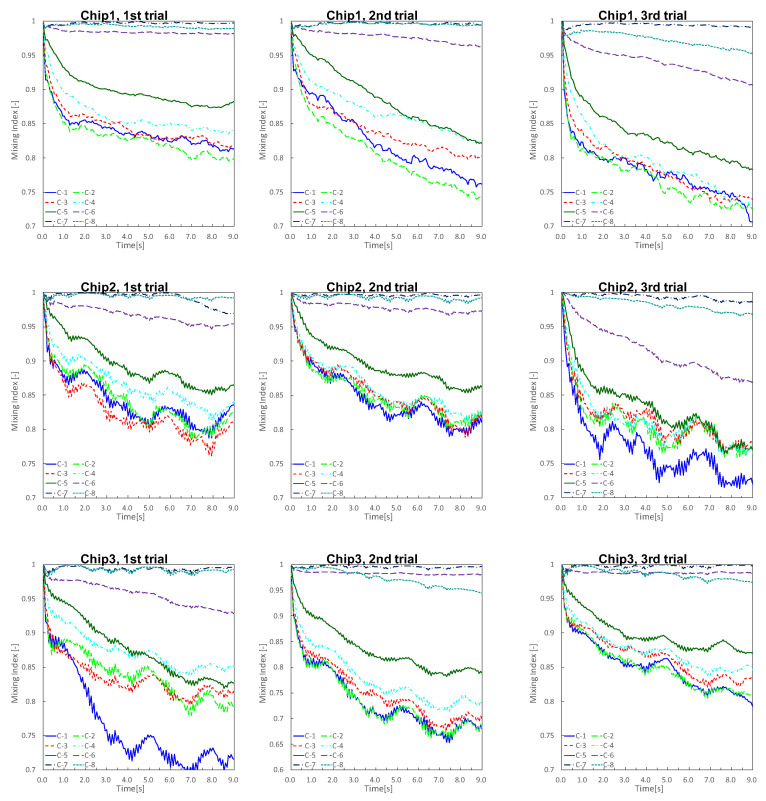
Time variation of mixing index until 9 s after application of air pressure. Graph was drawn using 3 points of a simple moving average. Mixing index indicates the degree of mixing; the lower the mixing index was, the more the beads were absorbed and mixed.

**Figure 8:**
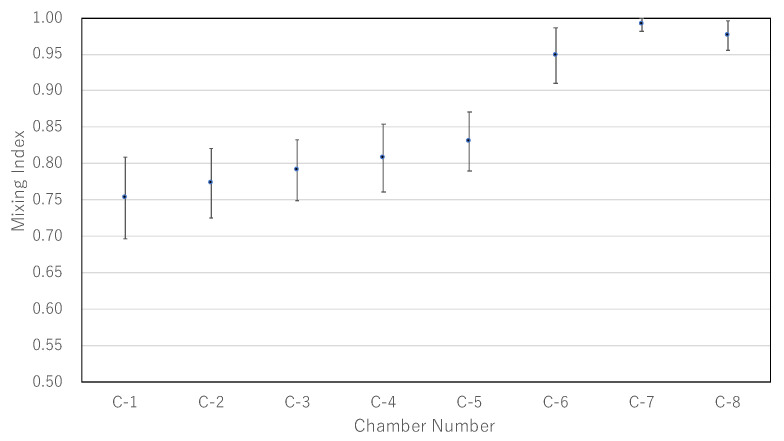
Graph of average of concentrations after 9 s for 9 experiments. Error bars represent standard error. The concentration gradient iwass generated roughly along the pressure gradient.

The experimental results are shown in Figure 6, where Figure 6a denotes the time variation of an experiment, and Figure 6b denotes the results obtained 9 s after the start of the experiment. The time variation of the concentration until 9 s after the application of air pressure is shown in Figure 7, where C-n indicates the n-th main chamber from the left. The raw data are very difficult to see, and the graph was drawn using 3 points of a simple moving average (raw data and why a simple moving average was applied are in Appendix B). The MI indicates the degree of mixing; the lower the MI is, the more the beads are absorbed and mixed. Similar results were obtained in all experiments, with a gradual decrease in concentration starting from the left chamber. Figure 8 is a graph showing the mean and standard error of MI after 9 s for the nine experimental results. The standard error of C-1 was large ( standard errors of C-1 and C-2 were 0.0199 and 0.169).

## 4. Discussion

### 4.1. Experiment Evaluation

**Figure 9:**
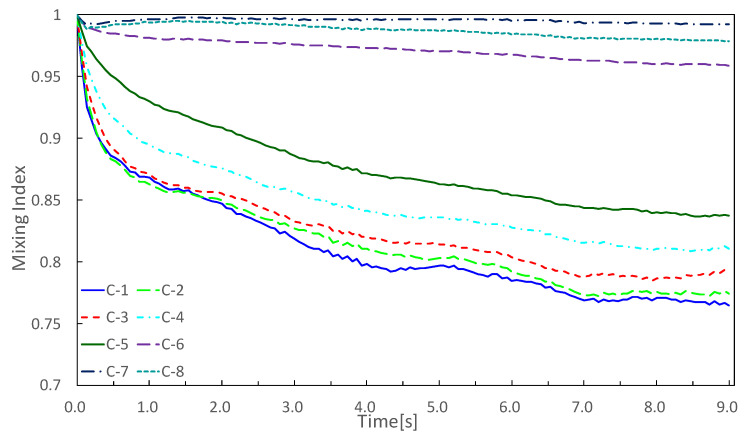
Average of nine experiments was calculated, and graph shows the simple moving average. Final concentrations were C-1, -2, -3, -4, -5, -6, -8, and -7.

In order to comprehensively check whether the concentration gradient was generated, the average value of the nine experimental results was calculated, and a simple moving average was applied, as shown in Figure 9.

Concentration gradients were generated along the pressure gradient, but chambers at both ends did not follow the pressure gradient; C-1 was almost the same absorption speed and mixing index as those of C-2, and C-8 was larger than the mixing index of C-7. This may have been caused by wall friction loss. Except for the chambers at both ends (C-1 and C-8), concentrations became thinner in the order of C-2, -3, -4, …, -7, and the concentration gradient was generated along the pressure gradient. However, there was a large difference in the mixing index between C-5 and C-6. A detailed design method for generating a uniform concentration gradient has not been established, and it is necessary to explore the design of the driving chamber for generating a uniform concentration gradient in the future.

On average, we were successful in generating concentration gradients, but the order of the concentration gradients varied in each. In the future, it is necessary to pursue reproducibility so that the same concentration gradient could be obtained in all experiments.

### 4.2. Experiments Using Colored Water

In a previous experiment, we used 3 μm microbeads to evaluate the concentration. To confirm that the same results could be obtained with liquid–liquid mixing, we conducted an experiment using colored water.

**Figure 10:**
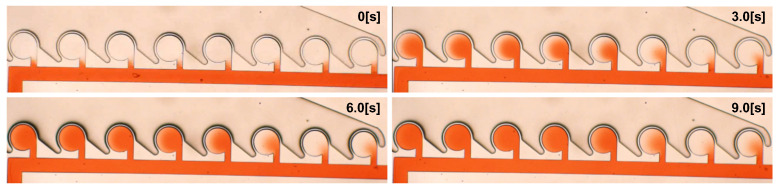
Time variation of experiment using colored water.

**Figure 11:**
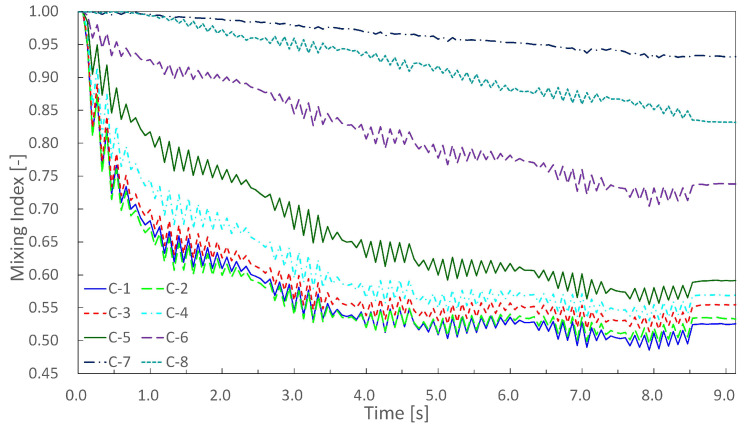
Time variation of mixing index in experiments using colored water. We obtained s similar result as that in experiments using 3 μm microbeads. Final mixing indices were C-1, -2, -3, -4, -5, -6, -8, -7.

The experimental results using colored water are shown in Figure 10 and Figure 11, where Figure 10 denotes the time variation in an experiment, and Figure 11 denotes the time variation in the mixing index. We obtained a similar result to that in the previous experiments.

### 4.3. Evaluation of Pressure Gradient

**Figure 12:**
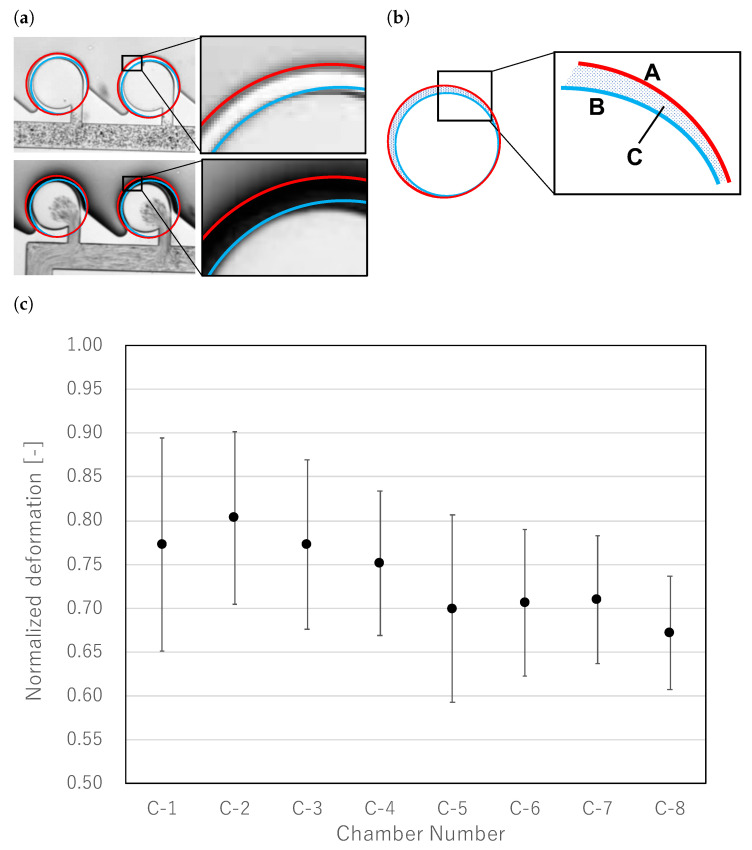
Evaluation of pressure gradient. (**a**) Wall deformation during expansion. Since the amount of deformation varied with the magnitude of pressure, it was used to evaluate the pressure gradient. (**b**) Details of evaluation region. Pixel values in Regions A and B were used to evaluate wall deformation in Region C. Equation (4) was used for evaluation. (**c**) Wall deformation in each chamber. Pressure gradients were generated approximately in the order of the concentration gradient.

The mixer generated a concentration gradient owing to the pressure gradient. To confirm that the mixer was functioning properly, we measured the deformation of the wall. The measurement method and results are shown in Figure 12, where Figure 12a,b denote the measuring area, and Figure 12c denotes the graph of the amount of wall deformation. When air pressure was applied, the wall between the driving chamber and the main chamber was significantly deformed (Figure 12a). Since the amount of deformation depends on the supplied pressure, we could evaluate the pressure gradient by comparing the amount of deformation. To measure the wall deformation, the main chamber was divided into three regions as shown in Figure 12b. Region A is the entire main chamber and the wall area, Region B is the main chamber area, and Region C is the wall area. Since the pixel value in Region C represents the amount of deformation of the wall, the pixel value in Region C was calculated using the pixel values in Regions A and B. The wall deformation was evaluated with the following formula;

(3) $$P_{X}\left(t\right)=\sum^{X}\;Pxl\left(t,x,y\right)$$

(4) $$ND=1-\frac{P_{C}\left(t_{expansion}\right)}{P_{C}\left(0\right)S_{C}}=1-\frac{P_{A}\left(t_{expansion}\right)-P_{B}\left(t_{expansion}\right)}{\left(P_{A}\left(0\right)-P_{B}\left(0\right)\right)\left(S_{A}-S_{B}\right)}$$

where ND denotes normalization deformation, *X* denotes each region, $P_{X}$ denotes the total pixel values of the region, Pxl(x,y) denotes the pixel value at x,y, and $S_{X}$ denotes the number of pixels of region *X*. Figure 12c shows the normalized deformation of the wall. It was confirmed that the pressure gradient was generally generated in the order of the concentration gradient. The standard error of C-1 was also larger than that of other regions (standard errors of C-1 and C-2 were 0.0431 and 0.348, respectively); therefore, as shown in Figure 7, the concentration of C-1 was unstable, so it was difficult to adjust the concentration according to the pressure gradient.

## 5. Conclusions

The flow path shown in Figure 6 was successful in generating a concentration gradient, but the chambers at both ends did not adhere to the gradient due to wall friction. If the goal is to generate the concentration gradient, the liquid in the chambers at both ends can be discarded, and the concentration gradient can be realized in the other main chambers. The volume of the main chamber of the channel designed in this study was 7.06 nL; therefore, even if the liquid in the chambers at both ends was discarded, the advantage of reducing the amount of liquid waste was not lost.

We attempted to generate a concentration gradient in an outer-circumference-driven mixer. We succeeded in generating a concentration gradient using the difference in the pressure applied to the driving chambers, which decayed as the distance from the air pressure source increased. As the micromixer was driven by air pressure, the channel could be expanded by increasing the applied pressure. However, although we succeeded in generating a concentration gradient, it was difficult to adjust the concentration in the main chambers according to the expected value (i.e., there was a large difference between C-5 and C-6). In the future, we aim to determine parameters that could fine-tune the concentration, and to further increase the scale of the system.

## Figures and Tables

**Figure A1:**
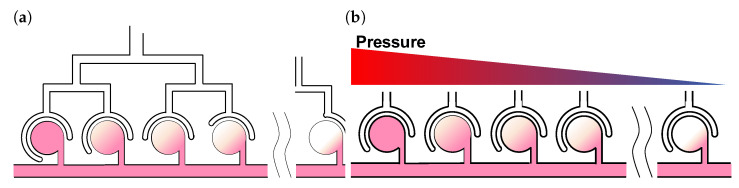
How to generate concentration gradient by outer-circumference-driven on-chip mixer. (**a**) Generation of concentration gradient by enclosure angle. Flow path was designed so that all driving chambers were equally pressurized, and the concentration was adjusted by enclosure angle. (**b**) Generation of concentration gradient by pressure gradient. All driving chambers had the same enclosure angle, and the concentration gradient was generated by the pressure supplied to the driving chambers.

**Figure A2:**
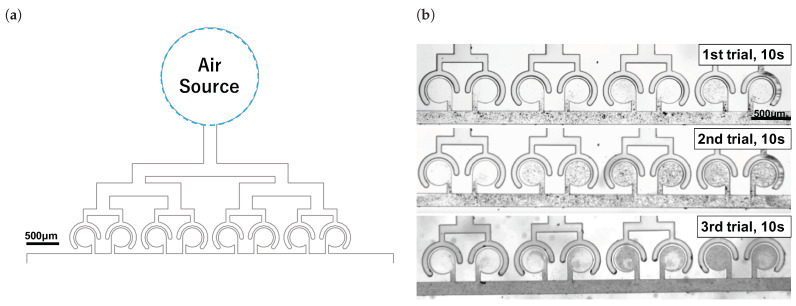
Verification of pressure uniformity. (**a**) To generate a concentration gradient using the enclosing angle, pressure must be uniform to generate a concentration gradient using the enclosing angle. (**b**) Experimental results. Concentrations were not uniform.

**Figure A3:**
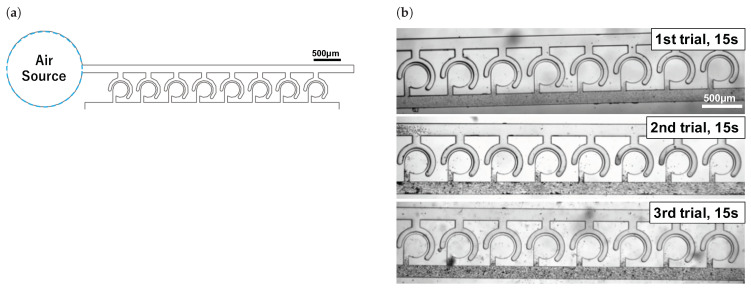
Use of pressure gradient. (**a**) Air was supplied from the left side of the figure, and the driving chamber was deformed by air pressure. The pressure gradient was generated by the pressure drop through the flow path, which generated the concentration gradient. (**b**) Experimental results of (**a**). Each chamber was driven for 15 s, but no stirring occurred.

**Figure A4:**
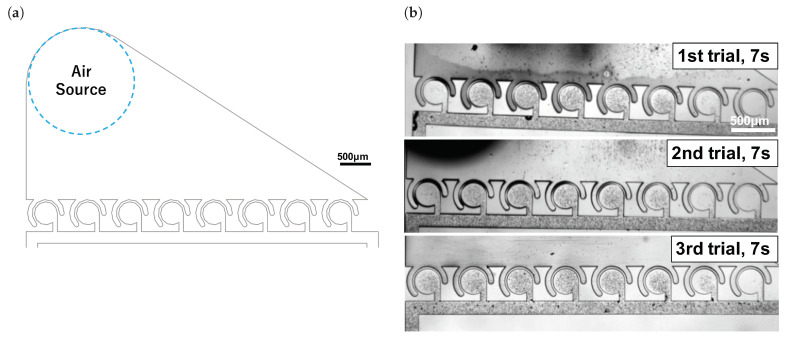
Flow path without the effect of channel width. (**a**) Concentration gradient was generated by the pressure gradient after removing the effect of the channel width. (**b**) Experimental results. Concentration gradient was generated, but concentrations in the chambers at both ends were low.

**Figure A5:**
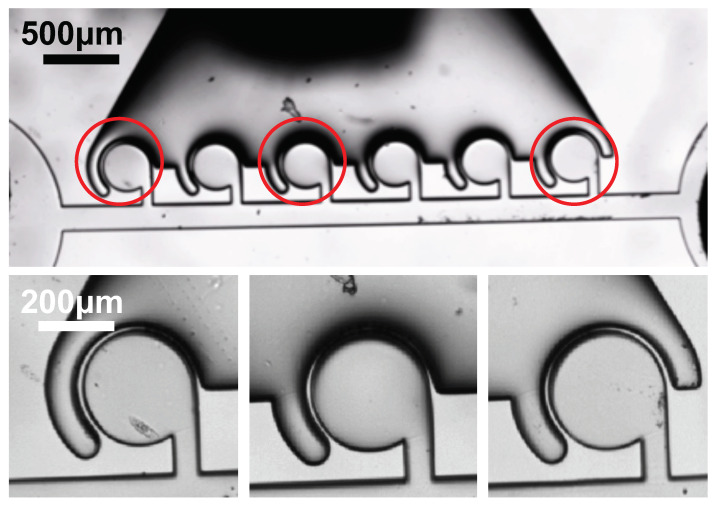
Wall deformation in relation to the position of the pneumatic source. Deformation differeed depending on the position of the chamber.

**Figure A6:**
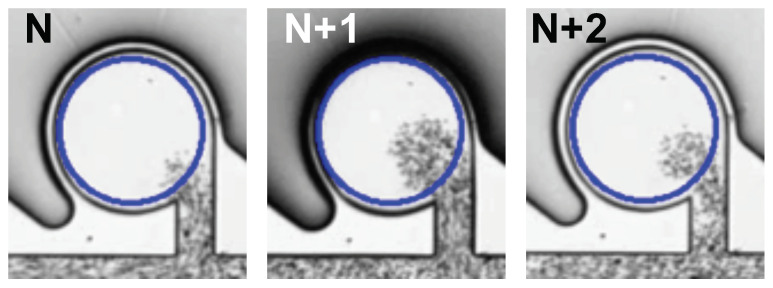
N-th to N + 2nd frame of an experiments. Main chamber repeatedly expanded and contracted, which caused rattling luminance values.

**Figure A7:**
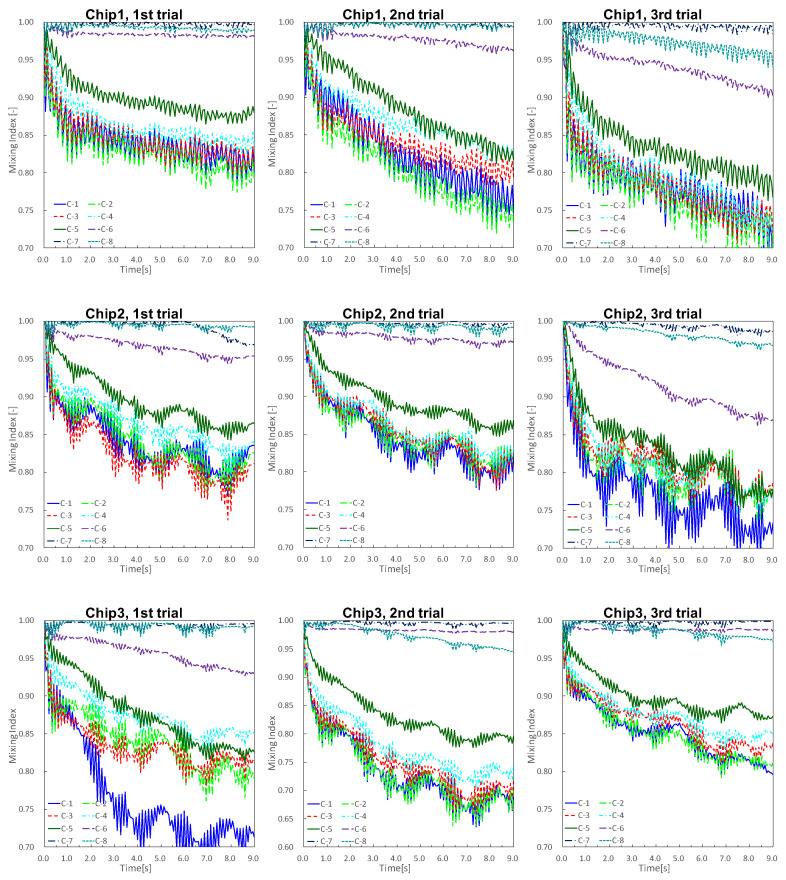
Time variation in mixing index until 9 s after the application of air pressure.

## Data Availability

The datasets used and/or analyzed during the current study are available from the corresponding author on reasonable request.

## Appendix A. Determination of Channel Design

### Appendix A.1. Concentration Gradient Generation Method

In this study, air was used. Therefore, the driving frequency cannot be increased owing to compressibility, but large deformation occurred in the driving chamber by increasing the driving pressure; therefore, the main chamber could be the same stirring speed as that in a previous study [32,33,34,35,36].

When an outer-circumference-driven mixer is driven using piezoelectric actuators, it was confirmed in previous studies [32,33,34,35,36] that the concentration in the main chamber could be adjusted depending on how much the driving chamber surrounds the main chamber (enclosure angle) and the pressure applied to the driving chamber. Therefore, there are two processes to generate the concentration gradient:

1. Equalize pressure in each driving chamber and adjust the concentration on the basis of enclosure angle.
2. Make the enclosure angle of each driving chamber uniform, and adjust the concentration on the basis of pressure.

The concept of each is shown in Figure A1. To investigate whether the concentration gradient using air could be realized in these channels, we designed a verification channel. As the first process requires uniform pressure supply, a linearly symmetrical branch channel was used to verify whether the concentration can be made uniform. In the second process, a serial-type channel was used to verify if a concentration gradient could be generated. If it was not possible to generate a concentration gradient, it was necessary to devise a new channel shape to drive the device on the basis of air pressure.

### Appendix A.2. Verification of Ability to Generate Chambers with Uniform Concentration Using Branch Channels

To generate a concentration gradient based on the enclosure angle, the pressure must be uniformly supplied to each driving chamber. To verify this, an experiment was conducted using the flow path shown in Figure A2a. The driving chambers were arranged independently, so that they were not affected by the neighboring chambers, and the driving chambers were designed in a treelike pattern, so that there was no difference in flow length among the driving chambers. If concentrations in all the main chambers were equal (Figure A2a), concentration could be adjusted by the enclosure angle. The experimental results are shown in Figure A2b. Concentrations in the main chambers are not uniform, and it is difficult to adjust them on the basis of enclosure angle. When pneumatic pressure is used, the pressure may not be evenly transmitted if the flow path is narrow. Therefore, the application of air pressure to such a branch channel was not appropriate to generate a concentration gradient.

### Appendix A.3. Generation of Concentration Gradients Based on Serial-Type Flow Paths

As mentioned in the previous section, it is difficult to evenly supply pressure to the driving chamber; therefore, we attempted to generate a concentration gradient based on pressure gradient. The flow path is shown in Figure A3a. The farther the distance from the air source, the more pressure loss occurs owing wall friction, and the more pressure gradient is generated in the driving chamber. The experimental results are shown in Figure A3b. No stirring occurred at all owing to the effect of the channel width, as described in the previous section.

Assuming that the channel width affects agitation, we designed the channel to generate the concentration gradient based on the pressure gradient by removing the effect of the channel width and conducted the experiment. The flow path shown in Figure A4a was designed in a way that the width of the flow path between the pneumatic source and each driving chamber was maximal. Experimental results are shown in Figure A4b. Although a concentration gradient was generated, the chambers located at both ends of the flow path exhibited a low concentration because of the pressure loss caused by wall friction.

The aforementioned results imply that it is difficult to supply pressure to the driving chamber as expected when the channel width of the driving chamber is narrow. Therefore, when air is used for pressure transmission and the frequency of expansion/contraction is high, it is better to increase the channel width of the driving chamber as much as possible.

### Appendix A.4. Influence of Deformation of Wall

Another possible reason for the diluted concentration in the chambers at both ends is the difference in the deformation of the driving chamber. Figure A5 shows how the wall is deformed when the driving chamber is driven using a high-speed camera. There is a difference in the deformation of the driving chamber depending on the positional relationship between the air pressure source and driving chamber. It is not clear whether this affects the concentration or not, but the parameters related to the concentration can be reduced by removing this effect.

## Appendix B. Raw Data of the Experiments

Figure A7 shows raw data of Figure 7. In Figure A7, the graph exhibits rattling behavior owning to the recording frame rate. Figure A6 shows the n-th, N + 1st, and N + 2nd frames in the experiments. The main chamber repeatedly expanded and contracted, which caused the liquid to be gradually absorbed as it moved back and forth between the neck chambers, resulting in rattling luminance values during the driving chamber operation.
